# Suicide‐Related Medical Claims, Downstream Healthcare Costs, and the Economic Impact of Enhanced Outpatient Services

**DOI:** 10.1111/sltb.70148

**Published:** 2026-11-04

**Authors:** Scott Graupensperger, Kate H. Bentley, Millard Brown, Adam Chekroud, Matt Hawrilenko

**Affiliations:** ^1^ Spring Health New York New York USA; ^2^ Yale School of Medicine, Psychiatry New Haven Connecticut USA

**Keywords:** employee assistance program, place of service, self‐harm, workplace mental health

## Abstract

**Objective:**

Suicide‐related medical claims are often followed by sustained healthcare utilization. This study quantified post‐crisis cost trajectories and examined whether coordinated outpatient mental health care was associated with lower downstream spending.

**Methods:**

We analyzed longitudinal medical claims from 13 employers. In Aim 1, individuals with a suicide‐related claim (*n* = 5574) were matched to demographically and clinically similar controls without such claims (*n* = 16,632). In Aim 2, individuals who used an employer‐sponsored mental health program within 1 month of a suicide‐related claim (*n* = 248) were matched to non‐users (*n* = 248).

**Results:**

Relative to matched controls, individuals with suicide‐related claims incurred more than double the monthly costs during the year following the suicide‐related claim, corresponding to adjusted excess of $547 per month ($6564 annually), not including the month of the index claim. Among those with a suicide‐related claim, early program engagement was associated with 37% lower monthly spending over Months 1–12 post‐claim ($720 less per month; $8637 annually).

**Conclusions:**

Suicide‐related claims are associated with sustained economic burden for employers and health plans. Early coordinated outpatient mental health care was associated with lower subsequent healthcare spending, especially spending in acute, high‐cost settings. These findings highlight the potential economic value of structured behavioral health services following suicide‐related claims.

## 1 Introduction

Suicide remains a leading cause of preventable death in the United States, but its burden extends well beyond mortality. Each year, millions of adults experience suicidal ideation or attempt suicide, often resulting in high‐acuity healthcare encounters that persist long after the immediate crisis (CDC 2024; SAMSHA 2023). Despite expanded national investment in prevention efforts, suicide rates have continued to rise (Gould et al. 2025; Martinez‐Ales et al. 2020; SAMHSA 2022). While the human toll is undeniable (Jordan and McIntosh 2011), the sustained healthcare utilization that follows suicide‐related crises represents an underexamined dimension of this public health challenge.

Nonfatal suicide attempts account for the vast majority of suicide‐related healthcare spending (Shepard et al. 2016). Suicidal ideation and behavior are frequently managed as high‐acuity conditions requiring intensive evaluation and stabilization in emergency departments and inpatient psychiatric settings (Zhdanava et al. 2021). Although such care, which may involve involuntary admission, is often clinically necessary (Bommersbach et al. 2024; Morris and Kleinman 2020), healthcare expenditures commonly remain elevated in the months following the crisis. Prior analyses have documented sustained post‐event cost elevations, though magnitude varies across study populations and methods (Pilon et al. 2022). Despite efforts to redirect crisis care toward lower‐intensity alternatives (Mancini et al. 2023), scalable approaches for delivering coordinated, cost‐conscious post‐crisis care remain limited.

Although suicide prevention initiatives are primarily evaluated based on clinical outcomes (Fox et al. 2020), there is growing interest in their economic impact. Several randomized trials have demonstrated that structured post‐crisis interventions can reduce recurrent suicidal behavior and generate substantial downstream healthcare savings (Bernecker et al. 2020; Dunlap et al. 2019; McCutchan et al. 2022; Park et al. 2018). However, these interventions have typically been tested in narrowly defined, high‐risk samples (e.g., military populations) and require intensive clinician training and fidelity monitoring, limiting their scalability and applicability to broader, real‐world populations experiencing suicide‐related crises.

From an employer and health plan perspective, the economic impact of suicide‐related care depends on both the cost structure and reach of available services. Scalable, technology‐enabled mental health platforms have emerged as a promising model for delivering rapid access to evidence‐based care while maintaining consistent quality and coordination across diverse populations (Attridge 2019; Langlieb et al. 2021; Ward et al. 2023). Prior evaluations suggest that such platforms can improve engagement, reduce fragmentation, and lower downstream healthcare spending (Bondar et al. 2022; Hawrilenko, Smolka, Ward, Ambwani, et al. 2025; Hawrilenko, Smolka, Ward, Kavelaars, et al. 2025; Ward et al. 2025). However, despite encouraging evidence of their clinical benefit for individuals screening positive for suicide risk (Graupensperger et al. 2025), there remains limited real‐world evidence on whether these platforms reduce healthcare spending following suicide‐related crises, leaving open the critical question of whether they can meaningfully bend the cost curve for high‐risk individuals.

### 1.1 Current Study

To address this gap, we leveraged large‐scale, longitudinal administrative claims data to quantify the healthcare cost trajectory surrounding suicide‐related medical claims and to test whether early engagement in a coordinated mental health platform is associated with attenuation of downstream spending. We analyzed medical claims from 13 employer clients of a comprehensive behavioral health benefit, representing more than 1.3 million covered lives and over 30 million member‐months of observation. The program's treatment engagement data (i.e., therapy, medication management) were integrated at the person‐month level with these claims to create a unified longitudinal dataset spanning 12 months before and after suicide‐related claims (25 months in total).

The study had two complementary aims. Aim 1 quantified the incremental healthcare costs associated with suicide‐related claims by comparing individuals experiencing such claims to demographically and clinically matched controls without suicide‐related claims. Aim 2 examined whether engagement with the mental health platform within 1 month of a suicide‐related claim was associated with divergence in post‐claim cost trajectories relative to matched non‐engagers. We hypothesized that individuals who engaged promptly following a suicide‐related claim would incur lower medical spending during the subsequent year, even when accounting for the costs of the program‐based care.

## 2 Methods

This study involved secondary analysis of deidentified administrative claims and program utilization data generated through routine clinical care and benefit administration, independent of research activities. The use of these data for research purposes is covered under an ongoing determination by the Yale University Institutional Review Board that such analyses do not constitute human subjects research.

### 2.1 Platform Overview

The program evaluated is a comprehensive, employer‐sponsored, technology‐enabled mental health platform offered as part of employee assistance benefits to employees and covered dependents (Graupensperger et al. 2025). The platform primarily provides access to psychotherapy and medication management delivered via video or in‐person formats by therapists with master's‐ or doctoral‐level licenses. Care is supported by master's‐level care navigators who assist with triage, provider matching, scheduling, benefits coordination, and follow‐up outreach as needed. Depending on the employer's benefit design, between 6 and 24 psychotherapy or medication‐management sessions are covered annually at no cost to the patient, and additional visits may be accessed through standard in‐network insurance benefits with applicable co‐payments. The underlying health insurance held by these members is standard employer‐sponsored coverage, and the program represents an additional benefit layered on top of it rather than a more generous insurance plan. Findings should therefore be interpreted as reflecting an enhanced behavioral health benefit within otherwise typical US employer insurance.

The program operates under a measurement‐based care framework, routinely collecting standardized symptom assessments to inform treatment planning, monitor progress, and guide clinical decision‐making (Bondar et al. 2022). The platform also incorporates advanced patient‐provider matching algorithms designed to optimize clinical fit and strengthen therapeutic alliance (Ward et al. 2026). As of May 2025, the program additionally offers optional AI‐augmented blended care to provide structured support between sessions, which may enhance treatment momentum and engagement (Graupensperger et al. 2026).

Importantly, access to the platform is voluntary. Employees and dependents covered under participating employer‐sponsored health plans (hereafter referred to as covered lives) must actively register with the platform to become a program member before receiving services. There is no automated referral pathway from external medical settings or electronic health records (EHRs) into the platform; enrollment occurs independently of medical claims activity.

### 2.2 Data Source and Cohort Construction

Administrative medical claims data from March 2020 through October 2025 were obtained from 13 employer‐sponsored health plans across diverse industries (e.g., manufacturing, retail, media). For each employer, claims data were included beginning 12 months prior to the employer's program launch date and extending through 3 months before the most recent available claim to allow for claims run‐out.

The analytic dataset included over 1.3 million covered lives and over 30 million participant‐month observations within the eligible study window. The 13 participating employers represented 6 industry sectors: manufacturing (*n* = 3, 68.0% of participants), healthcare (*n* = 4, 16.0%), biotechnology (*n* = 1, 6.1%), retail (*n* = 3, 4.1%), media (*n* = 1, 2.9%), and insurance (*n* = 1, 2.8%). The primary unit of analysis was the participant‐month. Suicide‐related claims were identified using an ICD‐10‐CM algorithm applied at the claim‐line level and aggregated to the participant‐month level. A participant‐month was classified as suicide‐related if any diagnosis position (up to 6 per claim line) contained codes indicative of suicidal ideation or intentional self‐harm. Qualifying codes included R45.851 (suicidal ideation) and multiple codes indicative of suicide attempt: T14.91x (suicide attempt, including encounter‐specific extensions), intentional self‐harm external cause codes (X71‐X83), and poisoning codes (T36‐T65) when accompanied by an intent modifier indicating intentional self‐harm (e.g., Bentley et al. 2024; Simon et al. 2022). This definition captures clinically meaningful suicide‐related encounters while maintaining accuracy when leveraging administrative claims data to define suicide‐related events.

Suicide‐related events were identified exclusively from external medical claims data rather than services delivered within the mental health program. Because program‐delivered services are not automatically written back into external medical records or claims databases, restricting case identification to external claims ensured a consistent and non‐overlapping data source. Participants were classified as suicide‐related cases if they had at least one qualifying suicide‐related claim during the eligible study period. The month of the first observed suicide‐related claim was defined as the index month. Two distinct matched comparison frameworks were then constructed to address the study's aims.

#### 2.2.1 Aim 1 Panel: Healthcare Costs Among Individuals With and Without Suicide‐Related Claims

To estimate the incremental healthcare cost associated with suicide‐related claims, each individual with a claim was matched to demographically and clinically similar controls without a suicide‐related claim during the study period. Matching procedures applied the nearest‐neighbor algorithm based on employer, age, gender, behavioral‐health diagnostic category, and log‐transformed medical risk scores derived from a hierarchical condition category (HHS‐HCC) risk adjustment model (Kautter et al. 2014). Behavioral health diagnostic category was derived from claims‐based ICD‐10‐CM F codes recorded as the primary mental health diagnosis in monthly claims aggregates. Participant‐months were flagged for mood (F3x), anxiety (F4x), substance use disorder (F1x), or other behavioral health diagnoses (other F codes). A single participant‐level diagnostic category was assigned hierarchically (Mood > Anxiety > SUD > Other > None) based on the presence of these flags at any point during the eligible observation window. Medical risk was measured using an actuarial risk score available in the monthly health plan membership file. The risk score reflects each individual's relative expected healthcare expenditure based on demographic characteristics and prior diagnoses, as estimated using a hierarchical condition category (HCC)‐based risk adjustment model. Because risk scores are right‐skewed, we used the natural logarithm of the risk score for matching to improve covariate balance and reduce leverage from extreme values. Exact matching was specified for employer, behavioral‐health category, and gender.

We evaluated matching ratios up to 1:5 and selected a 1:3 ratio based on optimal covariate balance and retention of cases with acceptable post‐match standardized mean differences across covariates (all ≤ |0.10|). Matching yielded 6518 individuals with a suicide‐related claim and 19,458 matched controls. Because some cases received fewer than three matched controls, inverse‐matching weights were applied to preserve the intended 1:3 case–control ratio in subsequent analyses. Analyses additionally required at least six observed person‐months within the relevant pre‐ or post‐index window and an available actuarial risk score. The resulting analytic sample comprised 5574 individuals with a suicide‐related claim and 16,632 matched controls (98.2% had 3 matches). For each matched control, a pseudo index month was assigned by sampling from the empirical distribution of index months observed among cases within the matched strata. This procedure preserved calendar‐time alignment between cases and controls and allowed construction of symmetric pre‐ and post‐index observation windows.

The Aim 1 panel had a mean age of 29.8 (SD = 13.3, range = 12–80), and 55.2% identified as female. Behavioral‐health diagnostic categories were 9.9% Mood, 7.4% Anxiety, 1.1% SUD, and 2.7% other (remainder were None). Eligibility records indicated that 53.0% of the Aim 1 panel were covered dependents and 35.9% were the employee of record, with status unavailable for 11.1%.

#### 2.2.2 Aim 2 Panel: Comparing Individuals With Suicide‐Related Claims Who Used the Program to Those Who Did Not

To evaluate the cost impact of program engagement, analyses were restricted to individuals with a suicide‐related claim and at least three observed months of follow‐up after the index claim. Among these, 249 (4.0%) used the mental health benefit program within 1 month of the suicide‐related index claim. Program engagement was defined as psychotherapy or medication‐management services within one calendar month of the suicide‐related index claim. Alternative engagement thresholds were evaluated in sensitivity analyses (e.g., multiple visits or extended follow‐up windows).

Similar to Aim 1, program users were matched to non‐users on employer, age, gender, behavioral health diagnostic category, and log‐transformed clinical risk score, with exact matching specified for employer, gender, and behavioral health category. In this aim, we additionally matched on average monthly healthcare spending during the 12 months preceding the index claim and on healthcare spending in the index month. Inclusion of these cost‐based covariates was intended to mitigate potential selection bias and account for baseline cost trajectory and acute severity at the time of the suicide‐related event. Note that the control group did not comprise strictly non‐treatment seekers, but rather covered lives who did not use the mental health benefit program under study during the defined exposure window.

We evaluated various matching ratios and selected a 1:1 specification because higher ratios materially degraded covariate balance and/or reduced the number of successfully matched program users. Using the 1:1 ratio, 248 of 249 program users were successfully matched to a non‐user, and post‐match standardized mean differences were < |0.10| across all covariates. The Aim 2 sample ultimately comprised the 248 program users and 248 non‐users who were matched on demographic and clinical risks (*N* = 496). This sample had a mean age of 35.2 (SD = 12.8, range = 13–67), was 49.6% female, and most were categorized as having mood (81.0%) or anxiety (13.3%) behavioral‐health diagnoses. For the Aim 2 cohort, 55.6% of participants were the employee of record rather than a covered dependent.

### 2.3 Medical Claims and Healthcare Costs

To reduce distortion from extreme non‐behavioral health‐related expenditures, participant‐months flagged for catastrophic chronic conditions (cancer, advanced chronic kidney disease, or end‐stage renal disease) were excluded. For each participant‐month, total medical costs (USD) were aggregated across all claims and truncated at $100,000 per month to limit the influence of outliers. For employers that reported only paid amounts, allowed costs were imputed using a standardized adjustment ($\text{allowed}=\frac{\text{paid}}{0.8}$). Program utilization costs, valued as amounts billed to the employer, were merged at the same monthly level from internal data tables, and summed to derive a composite cost variable that is inclusive of program treatment costs. That is, the cost of using the program was included in the total healthcare spending values. Because participants have claims data only for months in which they were covered by the health plan, cost outcomes were specified as the mean healthcare spending per observed participant‐month. Months without coverage contributed no observations rather than being treated as months of zero spending. All costs are therefore reported from the perspective of the employer and health plan sponsor.

### 2.4 High‐Cost Place‐of‐Service Spending

To explore potential mechanisms underlying post‐claim cost differences, we evaluated healthcare spending across select high‐cost places of service during Months 1–12 following the index suicide‐related claim in the Aim 2 matched cohort. Place‐of‐service categories were derived from standardized Centers for Medicare & Medicaid Services (CMS) place‐of‐service (POS) codes recorded on claim lines. We focused on acute, high‐cost settings defined using the following CMS POS codes: emergency department (POS 23), inpatient hospital (POS 21), ambulance (POS 41), and inpatient psychiatric facility or partial hospitalization (POS 51 and 52). These settings were selected a priori due to their association with acute care intensity and disproportionate contribution to downstream healthcare expenditures. Because POS is recorded at the claim‐line level whereas the primary cost outcome is constructed at the participant‐month level, spending was apportioned across settings by applying each setting's share of monthly claim‐line expenditures to the participant‐month total used in the primary analysis. This approach preserves the cost truncation applied throughout and ensures that setting‐specific components sum to the participant‐month total. Spending was aggregated into acute high‐cost POS and all other POS, and is expressed per person‐month observed to account for differences in follow‐up duration.

### 2.5 Statistical Analyses

Analyses were conducted on weighted person‐month panels covering 12 months before and after the index suicide‐related claim (−12 to +12, inclusive). Matching weights derived from cohort construction were applied such that matched controls contributed fractional weights reflecting their case–control ratio.

For Aim 1, comparing individuals with a suicide‐related claim to matched controls, weighted mean monthly cost trajectories with 95% confidence intervals were estimated descriptively. Period‐specific effects for the pre‐claim (−12 to −1), claim (0), and post‐claim (+1 to +12) intervals were estimated using negative binomial regression models with a log link to accommodate the positively skewed and overdispersed distribution of monthly healthcare cost data. Models included a random intercept for employer and adjusted for age, gender, behavioral health diagnostic category, and log‐transformed medical risk score. Models for the claim and post‐claim periods additionally adjusted for average monthly pre‐claim costs to account for baseline utilization and isolate acute and downstream cost changes associated with suicide‐related claims. Models used a log link whereby exponentiated coefficients represent adjusted cost ratios (CRs), corresponding to the relative difference in expected healthcare spending between groups. Adjusted CRs were translated into monthly and annualized dollar (USD) differences by applying each estimated ratio to the weighted mean cost among matched controls in the corresponding period.

For Aim 2, analyses were restricted to individuals with a suicide‐related claim using the matched 1:1 cohort of program users and non‐users. We applied the same three‐period negative binomial modeling structure, covariate adjustments, and weighting approach. Average pre‐claim monthly cost was included in claim‐month and post‐claim models to account for baseline differences in utilization. High‐cost POS‐specific spending was modeled using the same negative binomial structure and covariate adjustment as the primary cost models.

We did not adjust for recurrent suicide‐related claims in cost models because recurrence is a post‐index, potentially treatment‐affected outcome on the causal pathway between program engagement and expenditures. Adjusting for recurrence would therefore bias estimates of the total effect of program engagement. Additionally, prior validation research has demonstrated that many short‐interval “repeat” suicide‐related codes reflect re‐documentation of the same clinical event rather than independent events (Bentley et al. 2024).

As a robustness check for both aims, we estimated difference‐in‐differences models using weighted Poisson pseudo‐maximum likelihood estimation with a log link and two‐way clustered standard errors (participant and employer). When the parallel trends assumption is supported, this approach evaluates whether the change in spending from the pre‐ to post‐period differed between groups, thereby accounting for time‐invariant unobserved differences not fully captured by covariate adjustment.

## 3 Results

### 3.1 Aim 1 Results

The Aim 1 analytic sample included 5574 individuals with a suicide‐related claim and 16,632 matched controls. Among suicide‐related claims, the majority (~80%) were identified via suicidal ideation codes, with a smaller proportion reflecting suicide attempt or other intentional self‐harm‐related codes (~20%). Because individual claims may contain multiple concurrent diagnostic codes or intent specifications, these categories are not mutually exclusive and are presented as approximate ranges. In weighted negative binomial regression models (log link), individuals with a suicide‐related claim had higher adjusted monthly healthcare costs than matched controls in all three periods (Table 1). In the pre‐claim period (−12 to −1), costs were 26% higher (CR = 1.26, *p* < 0.001). Unsurprisingly, in the index claim month (0), costs were nearly 24‐fold higher (CR = 23.9, *p* < 0.001). Central to the first study aim, the post‐claim period (+1 to +12) remained more than double for those with a suicide‐related claim (CR = 2.23, *p* < 0.001), even when adjusting for pre‐index spending. Adjusted CRs translate these relative differences into meaningful absolute cost increments. Compared with matched controls, adjusted mean costs were $104 higher per month in the pre‐claim period (95% CI [$74, $137]), $10,157 higher in the index claim month (95% CI [$9180, $11,234]), and $547 higher per month in the post‐claim period (95% CI [$486, $613]).

**TABLE 1:** Mixed effects negative binomial regression models estimating differential monthly healthcare costs by suicide‐related claim status versus demographically and clinically similar matched controls.

Covariate	Pre‐index healthcare costs per month		Healthcare costs during index claim month		Post‐index healthcare costs per month	
	Cost ratio	95% CI	Cost ratio	95% CI	Cost ratio	95% CI
Suicide‐related claim (yes vs. no)	1.26	[1.18, 1.34]*	23.9	[21.72, 26.36]*	2.23	[2.09, 2.38]*
Actuarial risk score (log)	1.48	[1.45, 1.51]*	1.32	[1.28, 1.37]*	1.33	[1.30, 1.36]*
Age	1.02	[1.02, 1.02]*	1.01	[1.01, 1.02]*	1.01	[1.01, 1.02]*
Gender (ref: female)	0.76	[0.71, 0.80]*	0.88	[0.81, 0.96]	0.81	[0.76, 0.85]*
Behavioral health category (ref: mood)						
Anxiety	1.09	[0.96, 1.24]	1.66	[1.36, 2.02]*	1.06	[0.93, 1.20]
Substance use disorder	1.80	[1.40, 2.30]*	1.27	[0.87, 1.86]	1.40	[1.09, 1.79]
Other	1.35	[1.13, 1.61]	1.72	[1.30, 2.25]*	1.03	[0.86, 1.23]
None	0.65	[0.59, 0.71]*	0.90	[0.78, 1.04]	0.67	[0.61, 0.73]*
Average monthly costs pre‐index claim^a^	—	—	1.39	[1.33, 1.46]*	1.26	[1.22, 1.29]*

*Note:* Models include a random intercept for employer. Observations are weighted to reflect 3:1 matching of controls to cases; weights are carried into estimation. The matched sample comprised 22,206 participants (suicide‐related claim *n* = 5574; matched control *n* = 16,632). Models were estimated among participants with an available actuarial risk score (*n* = 19,940 for the pre‐index and index‐month models; *n* = 17,833 for the post‐index model).

^a^ Rescaled to per $1000.

* *p* < 0.001.

Descriptively, individuals with a suicide‐related claim exhibited modestly elevated healthcare spending prior to the index event, a dramatic cost spike in the month of the event, and persistently higher costs throughout the subsequent year. The $547 additional spending per month corresponds to $6564 in excess expenditures over the 12 months following the suicide‐related claim, excluding the index month and adjusting for baseline spending patterns. Figure 1 displays the corresponding weighted cost trajectories centered on the index claim month, illustrating the modest pre‐index elevation, pronounced index‐month spike, and sustained post‐index cost excess.

**FIGURE 1:**
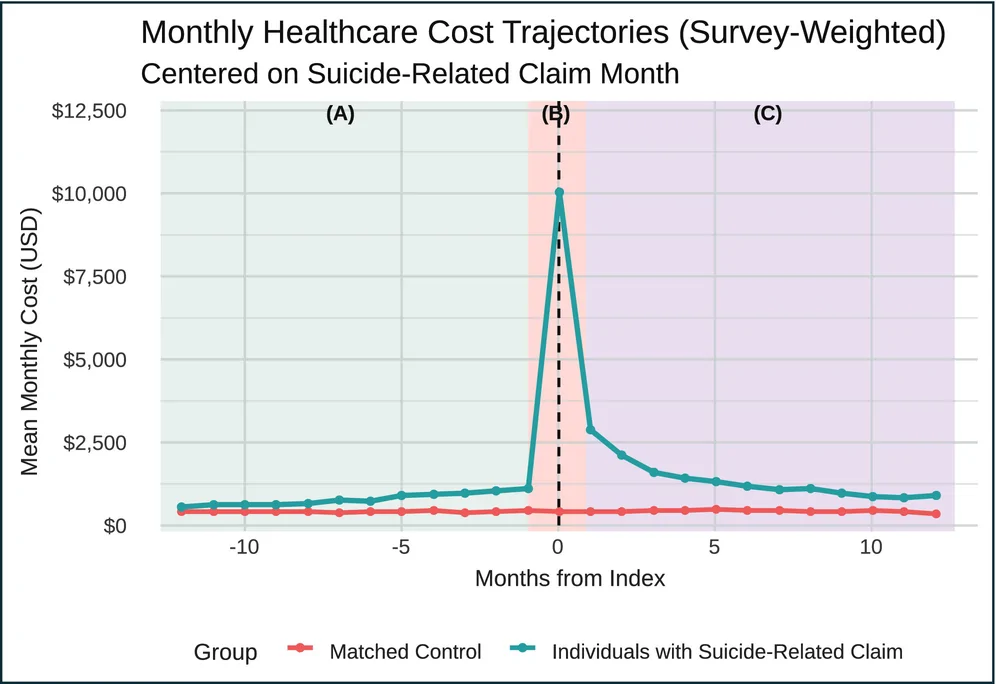
Monthly healthcare cost differences by period (weighted descriptive means). The matched control is those with similar demographic and clinical characteristics but did not have a suicide‐related claim. Error bars around point estimates are 95% confidence intervals (CI). Shaded areas indicate analysis windows: (A) Pre‐Index Δ/month: $104 [CI: $74, $137], (B) Index Claim Month Δ: $10,157 [CI: $9180, $11,234], (C) Post‐Index Δ/month: $547 [CI: $486, $613].

A complementary difference‐in‐differences model yielded a ratio‐of‐ratios of 1.66 [1.51, 1.82] (*p* < 0.001). This estimate should be interpreted with caution; however, the parallel‐trends assumption was not satisfied. Across the 12 months preceding the index claim, mean monthly costs among individuals with a suicide‐related claim rose from $442 to $1066, while costs among matched controls remained essentially unchanged ($421–$439). The difference in monthly trend was statistically significant (ratio per month = 1.07 [1.05, 1.09]; *p* < 0.001). This pre‐index divergence is an interesting finding in itself, suggesting that suicide‐related events may emerge within a trajectory of escalating healthcare utilization. The Aim 1 estimate therefore reflects both this escalating trajectory and the acute event.

### 3.2 Aim 2 Results

The Aim 2 sample included individuals with a suicide‐related claim who engaged with the program within 1 month (*n* = 248) and matched non‐users (*n* = 248). Recurrence of suicide‐related claims during follow‐up was low and did not differ significantly between groups (16.1% program users vs. 21.8% matched controls; OR = 0.69, *p* = 0.111). Weighted descriptive monthly cost trajectories are shown in Figure 2, and model estimates appear in Table 2.

**FIGURE 2:**
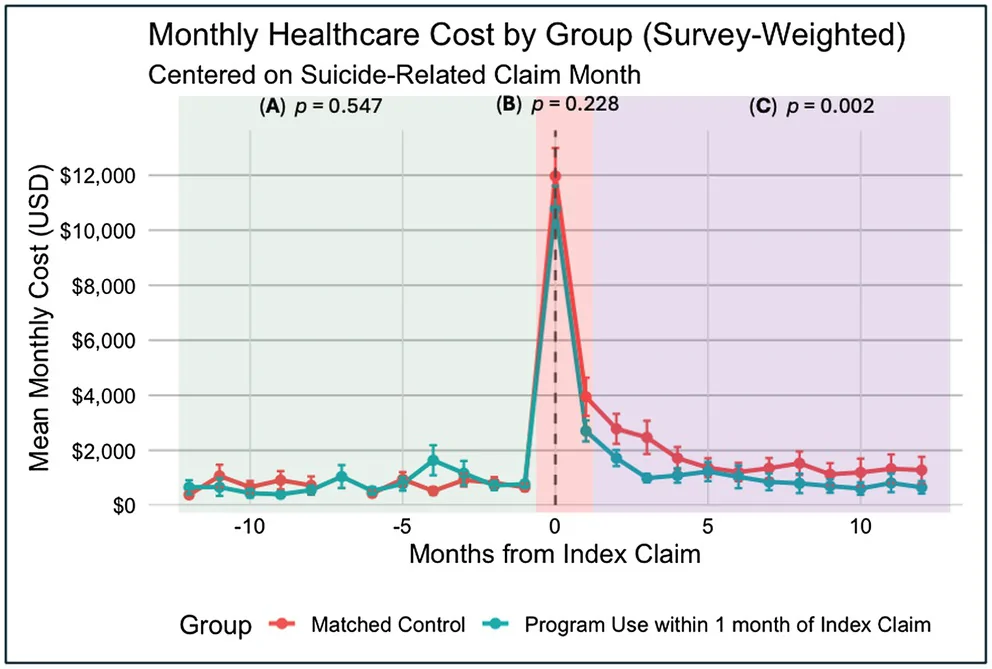
Monthly healthcare cost differences by period (weighted descriptive means). The matched control are those with similar demographic and clinical characteristics who had a suicide‐related claim but did not use the program within 1 month of the claim event. Error bars around point estimates are 95% confidence intervals (CI). Shaded areas indicate analysis windows: (A) Pre‐SI Δ/month: −$74 [CI: −$374, $141], (B) SI Claim Month Δ: $1425 [CI: −$987, $3388], (C) Post‐SI Δ/month: $720 [CI: $304, $1030].

**TABLE 2:** Negative binomial regression models estimating differences in monthly healthcare costs among those with a suicide‐related claim by program use within 1 month of claim versus demographically and clinically similar matched non‐users.

Covariate	Pre‐index healthcare costs per month		Healthcare costs during index claim month		Post‐index healthcare costs per month	
	Cost ratio	95% CI	Cost ratio	95% CI	Cost ratio	95% CI
Program user (vs. matched non‐user)	1.11	[0.79, 1.55]	0.88	[0.72, 1.08]	0.63	[0.47, 0.84]**
Actuarial risk score (log)	1.34	[1.18, 1.53]***	1.13	[1.04, 1.22]**	1.22	[1.10, 1.36]***
Age	1.02	[1.01, 1.04]**	1.01	[1.00, 1.01]	0.99	[0.98, 1.01]
Gender (ref: female)	0.74	[0.51, 1.05]	1.19	[0.96, 1.48]	0.95	[0.69, 1.29]
Behavioral health category (ref: mood)						
Anxiety	0.65	[0.37, 1.16]	0.72	[0.53, 0.99]*	0.68	[0.44, 1.04]
Substance use disorder	0.78	[0.34, 1.78]	0.83	[0.51, 1.35]	0.69	[0.36, 1.34]
None	0.22	[0.03, 1.44]	0.73	[0.23, 2.31]	0.18	[0.04, 0.88]*
Average monthly costs pre‐index claim^a^	—	—	1.09	[1.02, 1.16]**	1.15	[1.08, 1.22]***

*Note:* Observations are weighted to reflect 1:1 matching of controls to those with program engagement; weights are carried into estimation. Total *N* = 496 (Program users *n* = 248; Matched non‐users *n* = 248).

^a^ Rescaled to per $1000.

* *p* < 0.05.

** *p* < 0.01.

*** *p* < 0.001.

Prior to the claim, monthly costs were comparable between groups (CR = 1.11, *p* = 0.547), and no significant difference was observed in the claim month (CR = 0.88, *p* = 0.228). However, in the 12 months after the claim, program users incurred 37% lower monthly costs than matched controls (CR = 0.63, *p* = 0.002), corresponding to $720 lower spending per month (95% CI [$304, $1030]) for approximately $8637 lower total annual spending. Important to note is that these estimates are from a matched sample with matching based on both pre‐index costs as well as costs from the index month itself, making the estimate conditional on equivalent prior spending patterns, thereby isolating divergence in post‐claim cost trajectories.

As a robustness check using a participant fixed‐effects difference‐in‐differences specification, we estimated a ratio‐of‐ratios of 0.56 [0.38, 0.83] (*p* = 0.004), corresponding to model‐implied monthly savings of $849 [$330, $1202]. This alternative within‐participant approach yielded an effect that was directionally consistent and slightly larger in magnitude than the primary negative binomial model, reinforcing the finding of sustained post‐claim cost divergence among program users relative to matched controls (Figure 3). The parallel‐trends assumption was supported, as month‐by‐month estimates across the 12 pre‐index months showed no systematic divergence between groups (difference in monthly trend: ratio per month = 1.05 [0.95, 1.15], *p* = 0.340). One of 11 pre‐index months differed at *p* = 0.047, but consistent with Roth (2022), we note that pre‐trend tests have limited power and interpret this as supportive rather than confirmatory.

**FIGURE 3:**
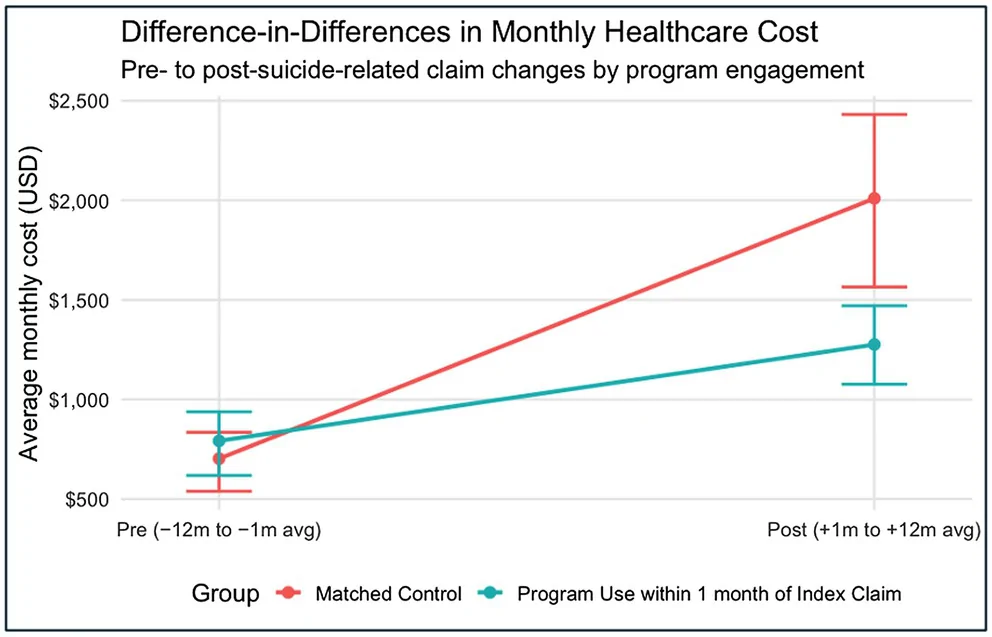
Difference‐in‐differences model estimates of average monthly cost before and after the suicide‐related index claim. Estimates derived from Poisson pseudo‐maximum likelihood models with participant fixed effects and cluster‐bootstrapped 95% confidence intervals.

### 3.3 High‐Cost Place of Service Spending

To evaluate a plausible driver of the observed post‐index cost reductions, we apportioned monthly healthcare expenditures by place of service during Months 1–12 following the index suicide‐related claim, focusing on emergency department, inpatient hospital, ambulance, and inpatient psychiatric or partial hospitalization settings (Figure 4). Spending in these acute high‐cost settings averaged $808 per person‐month among matched controls versus $534 among program users, a difference of $274 accounting for approximately 40% of the unadjusted difference in total monthly spending. In adjusted models, spending in these settings was significantly lower among program users (CR = 0.44 [0.20, 0.95]; *p* = 0.037), whereas the difference in spending across all other places of service did not reach significance (CR = 0.79 [0.60, 1.03]; *p* = 0.085). Together, these findings suggest that reduced spending in acute, high‐cost settings may partially, but not fully, explain the observed post‐index cost reductions.

**FIGURE 4:**
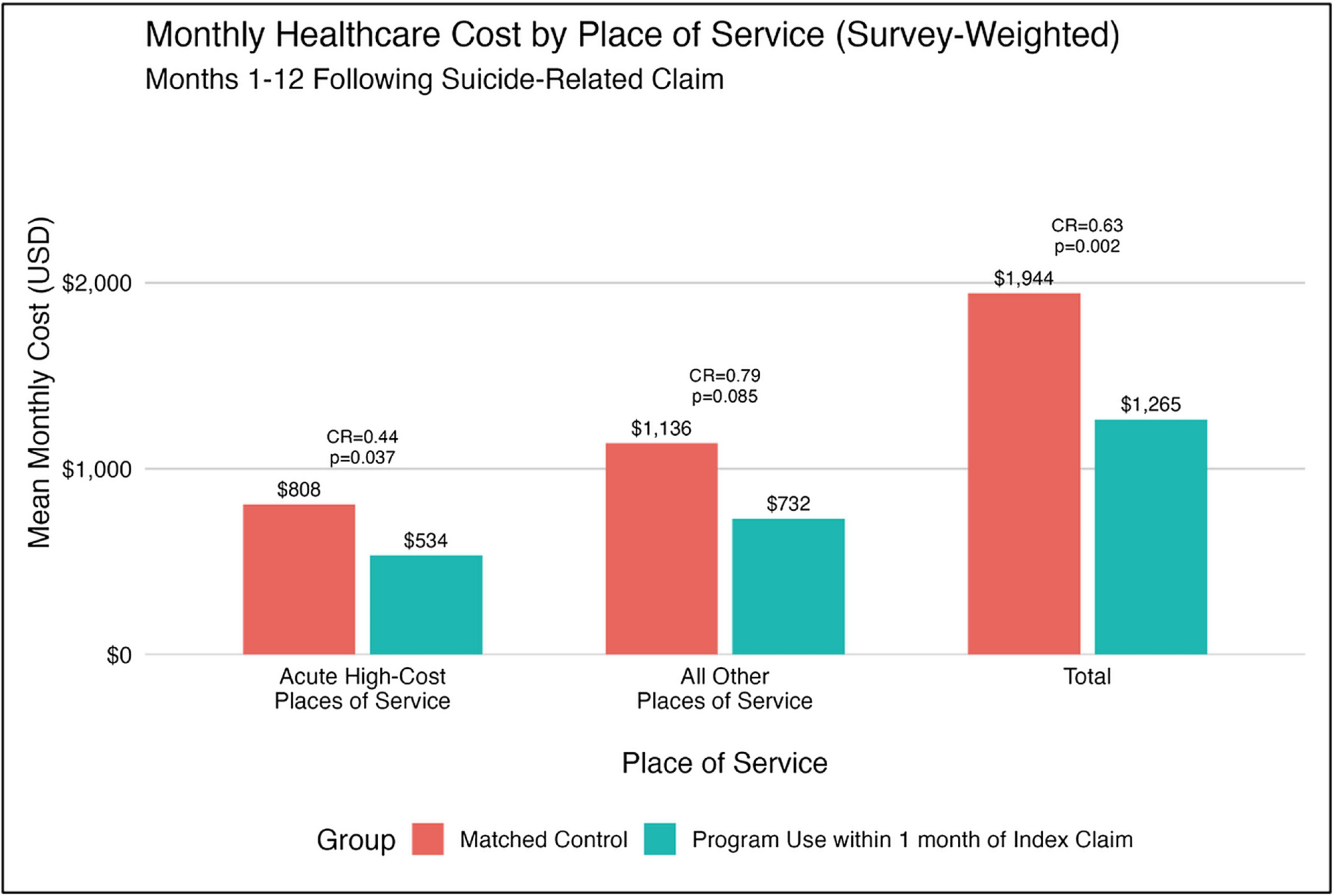
Mean healthcare spending per person‐month observed during Months 1–12 after the index suicide‐related claim. Acute high‐cost places of service comprise emergency department, inpatient hospital, ambulance, and inpatient psychiatric or partial hospitalization claims (CMS place‐of‐service codes 21, 23, 41, 51, and 52). Spending was apportioned by applying each setting's share of monthly claim‐line expenditures to the participant‐month total used in the primary analysis. Cost ratios (CR) and *p*‐values are from negative binomial models adjusted for age, log risk score, gender, behavioral health category, and pre‐index spending. *N* = 496 (248 program users; 248 matched non‐users).

### 3.4 Sensitivity Analyses

Findings were robust across multiple sensitivity analyses. We found no evidence of age‐based effect modification (i.e., interactions) when age was modeled continuously or categorically, indicating consistent cost effects across adolescents and adults. Because 44.8% of program users had engaged with the platform in the 12 months preceding the index event, we adjusted for prior use in sensitivity analyses. Inclusion of prior program use as a covariate did not attenuate the observed association between program engagement and post‐index costs (CR = 0.57; *p* = 0.001), and prior use itself was not significantly associated with downstream expenditures (CR = 1.26; *p* = 0.273).

Consistent with prior work distinguishing treatment initiation from sustained engagement (Bahraini et al. 2022), we examined alternative program utilization definitions requiring either ≥ 1 visit or ≥ 2 visits within the initial follow‐up window. Both operationalizations yielded directionally similar and statistically significant reductions in post‐index costs, and the estimates from the two definitions were not statistically distinguishable from one another (CR = 1.10, *p* = 0.681). These findings suggest that the observed cost differences may be primarily driven by treatment initiation rather than early visit volume or short‐term retention. Because observed follow‐up was somewhat shorter among program users (mean 10.5 vs. 11.3 months), we repeated the analysis restricted to the first four post‐index months, during which both groups were observed at comparable rates (98%–100%). The CR was 0.55 [0.40, 0.75], consistent with the full‐year estimate. Results were also consistent under alternative cost truncation thresholds and modeling specifications, including the fixed‐effects difference‐in‐differences framework described above.

## 4 Discussion

The primary finding from Aim 1 is that individuals with a suicide‐related medical claim incurred, on average, an additional $6564 in healthcare spending over the following year compared with matched controls, after adjustment for pre‐index spending patterns and other cost‐driving covariates (unadjusted difference was $10,926). In Aim 2, among individuals with a suicide‐related claim, those who engaged with the mental health program within 1 month experienced 37% lower post‐claim spending than matched non‐users, corresponding to approximately $8637 less in annual healthcare costs, even after accounting for program utilization and prior spending. Together, these findings highlight the sustained financial burden of suicide‐related events and the potential for timely connection to coordinated care to attenuate downstream costs. Viewed through an employer and health plan lens, suicide‐related claims may represent a critical inflection point that signals both acute clinical risk and heightened future medical expenditure.

The Aim 1 findings regarding the cost burden surrounding a suicide‐related medical claim are perhaps unsurprising, yet critical to document. Cost trajectories shown in Figure 1 demonstrated gradually rising healthcare utilization across the year preceding the index claim, particularly in the months immediately before the event. This pattern supports that suicide‐related claims often emerge within a broader context of escalating medical and behavioral health complexity rather than as isolated episodes (Fleury et al. 2025; Sakai‐Bizmark et al. 2022). Identifying such pre‐crisis utilization patterns may offer opportunities for earlier outreach or prevention strategies based on administrative health data (Simon et al. 2016). As expected, spending surged dramatically in the index month, consistent with acute care delivered in emergency or inpatient settings (Shepard et al. 2016; Zhdanava et al. 2021). Importantly, costs did not normalize following the crisis. Even after adjusting for baseline spending, healthcare expenditures remained elevated throughout the subsequent year. The magnitude of this sustained excess is comparable to the annual healthcare costs associated with major chronic conditions such as heart failure (~$8200; Zheng et al. 2024), highlighting the lasting economic impact of suicide‐related events.

In Aim 2, early engagement with the mental health program following a suicide‐related claim was associated with substantially lower downstream costs, even after accounting for program‐related care and pre‐index spending. Among individuals with comparable baseline and index‐month expenditures, those who engaged within 1 month incurred much lower monthly costs over Months 1–12 post‐index, relative to matched non‐users. Because matching incorporated both pre‐index and index‐month costs, these estimates reflect divergence in post‐claim cost trajectories among individuals with similar acute severity at the time of the event. Complementary difference‐in‐differences analyses yielded directionally consistent and slightly larger effects, reinforcing that the observed cost reductions were unlikely to be driven by pre‐existing spending trends alone.

To our knowledge, this is the first study to examine whether engagement with an employer‐sponsored mental health program following a suicide‐related claim is associated with subsequent changes in healthcare spending. The observed cost attenuation among early program users is consistent with a coordinated‐care mechanism including rapid access to evidence‐based therapy and medication management, active care navigation that supports treatment adherence and continuity, and measurement‐based follow‐up that enables timely intervention if symptoms worsen (Chekroud 2018; Simon et al. 2018). By facilitating structured outpatient care during a high‐risk period, such programs may help stabilize individuals after a crisis and reduce reliance on acute, high‐cost services. In this way, post‐crisis engagement may not simply add behavioral health services, but alter the trajectory of broader healthcare utilization. Lower spending in this context is also unlikely to reflect withdrawal from care. Prior work examining this same program found that individuals with suicidal ideation who received crisis outreach showed improved treatment engagement and symptom improvement over 6 months of follow‐up (Graupensperger et al. 2025). Standardized clinical assessments are collected only for program users, however, and are therefore unavailable for matched non‐users, so clinical outcomes could not be evaluated alongside spending in the present analysis.

Employer‐sponsored mental health programs have previously been associated with reduced medical spending among workforce populations (Bondar et al. 2022; Hawrilenko, Smolka, Ward, Ambwani, et al. 2025; Penev et al. 2023). The present findings extend this evidence to the context of suicide‐related care, a setting characterized by particularly high short‐term medical expenditures. One plausible explanation for the observed cost divergence is reduced reliance on acute, high‐cost settings. Spending in the high‐cost settings examined accounted for approximately 40% of the total difference, indicating that reduced acute‐care spending contributes meaningfully to, but does not fully account for, lower downstream costs among program users. The remainder was distributed across other places of service, consistent with a broader reduction in healthcare utilization rather than substitution away from acute care alone (Olfson et al. 2025).

### 4.1 Implications for Employers and Payers

Suicide‐related crises are associated with sustained, elevated healthcare spending both in the immediate aftermath and throughout the subsequent year, highlighting their financial implications for employers and health plan sponsors. The finding that early engagement with a structured mental health program following a suicide‐related claim is associated with lower downstream medical spending has important implications for benefit design. Programs that facilitate rapid connection to coordinated outpatient services, including timely therapy, active care navigation, and measurement‐based follow‐up, may help reduce reliance on high‐cost acute settings during a vulnerable period. For payers and employers seeking to address rising behavioral health expenditures, these findings suggest that improving access to and uptake of coordinated post‐crisis care may represent a potentially cost‐relevant strategy (Hawrilenko, Smolka, Ward, Kavelaars, et al. 2025).

At the same time, program reach remains limited. Among covered lives with a suicide‐related claim, only 4% engaged with the program within 1 month of the index event. Although some likely sought mental health care outside of the program, this estimate aligns with national data demonstrating low rates of outpatient follow‐up after suicidal crises (Action Alliance for Suicide Prevention 2024). Indeed, even when employers offer comprehensive behavioral health benefits, activation and uptake of these services can be a critical gap. Strategies to increase engagement may include proactive claims‐based outreach, coordination with hospital discharge planning, and collaboration with occupational health and employee assistance programs, alongside efforts to reduce stigma and logistical barriers to care (Mann et al. 2021).

### 4.2 Strengths and Limitations

This study leveraged longitudinal administrative claims data from over 1.3 million covered lives across multiple employers, enabling examination of healthcare cost trajectories before and after suicide‐related medical claims in a large, real‐world population. The matched cohort design, employer‐level clustering, and adjustment for pre‐index spending patterns and clinical risk factors reduce many common sources of confounding in observational cost analyses. The use of complementary modeling approaches including weighted negative binomial regression and participant‐level difference‐in‐differences specifications, together with formal assessment of the assumptions underlying the latter, strengthens confidence in the robustness of the findings. Additionally, linkage of employer‐sponsored program utilization with external medical claims allowed for evaluation of cost patterns associated with program engagement in a high‐acuity clinical context in which longitudinal data collection is very challenging due to poor compliance (Schatten et al. 2025).

Several limitations should be considered. First, engagement with the mental health program was voluntary; therefore, unmeasured factors such as help‐seeking motivation, social support, or nuanced aspects of crisis severity may have influenced participation. Although matching incorporated demographic characteristics, diagnostic categories, actuarial risk scores, and both pre‐index and index‐month expenditures, residual confounding cannot be fully excluded. Second, suicide‐related events were identified using ICD‐10 codes within administrative medical claims, and not program‐based assessment as this would only be available to program users and not their matched controls. Suicidal ideation and behaviors are frequently under‐assessed, underreported, and incompletely captured in claims data (Peterkin et al. 2025); thus, our identification strategy likely represents a conservative subset of all suicide‐related events. Such misclassification would be expected to bias results toward the null, as some individuals categorized as controls may have experienced undocumented suicide‐related encounters or unrecorded symptoms. Relatedly, mortality could not be ascertained in these data nor could the reason behind a loss of healthcare coverage (e.g., changed jobs). Because participants contribute only observed months, any loss of coverage during the follow‐up period would truncate observation rather than register as reduced spending. Third, the Aim 2 sample was modest in size, which may limit precision and reduce power to detect differences in spending within individual places of service, particularly settings used by a small proportion of participants. Finally, data were drawn from 13 employers and their dependents, which may limit generalizability to other populations, including uninsured individuals or those covered by public insurance programs. The employer mix was also concentrated, with manufacturing accounting for 68.0% of participants, though we do not have access to the nature of the individual participant's role or participant within their employer.

### 4.3 Conclusions

Suicide‐related medical claims are uncommon but associated with sustained excess healthcare spending that extends well beyond the index crisis. In this multi‐employer, longitudinal claims analysis, individuals with suicide‐related claims experienced persistently higher post‐index costs, even after adjustment for baseline spending patterns. Among those with such claims, early engagement with a coordinated mental health program was associated with substantially lower downstream expenditures over the subsequent year. These findings suggest that suicide‐related events represent not only a clinical inflection point but also a period of heightened financial vulnerability for employers and health plan sponsors. Timely connection to structured outpatient care following a crisis may help stabilize individuals during this high‐risk period and reduce spending in acute, high‐cost settings. As employers and payers confront rising behavioral health expenditures, strategies that improve post‐crisis engagement in coordinated care warrant further consideration as a potentially cost‐relevant component of comprehensive suicide prevention efforts.

## Author Contributions

**Scott Graupensperger:** conceptualization, methodology, investigation, writing – original draft, writing – review and editing, visualization. **Kate H. Bentley:** conceptualization, writing – original draft, writing – review and editing. **Millard Brown:** conceptualization, supervision, writing – original draft, writing – review and editing. **Adam Chekroud:** resources, supervision, writing – original draft, writing – review and editing. **Matt Hawrilenko:** conceptualization, methodology, investigation, supervision, writing – original draft, writing – review and editing.

## Funding

No external funding was received.

## Disclosure

*Permission to Reproduce Material*: No previously published material requiring permission was reproduced in this manuscript.

*Clinical Trial Registration*: This study was not a clinical trial and was not prospectively registered.

## Ethics Statement

This study involved secondary analysis of deidentified administrative claims and program data generated through routine care. Under an ongoing determination by the Yale University Institutional Review Board, such analyses do not constitute human subjects research; therefore, institutional review board approval was not required.

## Consent

Informed consent was not required because the study involved secondary analysis of deidentified administrative data and did not involve direct participant contact.

## Conflicts of Interest

All authors report being employed by and holding equity in Spring Care Inc. outside the submitted work. Dr. Chekroud reports being the inventor on three patent submissions relating to treatment for major depressive disorder (US Patent and Trademark Office number Y0087.70116US00 and provisional application numbers 62/491660 and 62/629041) outside the submitted work. Dr. Chekroud further reports holding equity in Carbon Health Technologies Inc., Wheel Health Inc., Parallel Technologies Inc., Healthie Inc., and UnitedHealthcare; receiving consulting fees from Fortress; and providing unpaid advisory services to health care technology startups outside the submitted work. Dr. Bentley reported holding equity in and receiving consulting fees from Visible Health Inc.

## Data Availability

The data underlying this study were obtained under data use agreements with participating employers and contain sensitive administrative claims information; therefore, they are not publicly available.
